# What Really Lurks in the Dark? Revisiting the Occurrence of *Tomicus destruens* (Coleoptera, Curculionidae, Scolytinae) in Greece

**DOI:** 10.3390/insects17060579

**Published:** 2026-06-01

**Authors:** Dimitrios N. Avtzis, Hugo Mas, Matteo Bracalini, Achilleas Kaltsidis, Eleni I. Koutsogeorgiou, Stefanos S. Andreadis, Nikoleta Eleftheriadou, Massimo Faccoli

**Affiliations:** 1 Forest Research Institute, Hellenic Agricultural Organization «Dimitra», 57006 Vassilika, Greece; achilleas.kaltsidis@gmail.com (A.K.); neleftheriadou@elgo.gr (N.E.); 2 Laboratori de Sanitat Forestal, Centro para la Investigación y Experimentación Forestal de la CV, VAERSA, Conselleria de Agricultura, Generalitat Valenciana, Av. Comarques del País Valencià 114, 46930 Valencia, Spain; lab_plagas.ctv@gva.es; 3 Department of Agriculture, Food, Environment and Forestry, University of Florence, Piazzale delle Cascine, 18, 50144 Florence, Italy; matteo.bracalini@unifi.it; 4 Laboratory of Applied Zoology and Parasitology, School of Agriculture, Aristotle University of Thessaloniki, 54124 Thessaloniki, Greece; 5 Institute of Plant Breeding and Genetic Resources, ELGO Dimitra, 57001 Thermi, Greece; ekoutsogeorgiou@gmail.com (E.I.K.); sandreadis@elgo.gr (S.S.A.); 6 Department of Agronomy, Food, Natural Resources, Animals and the Environment, University of Padua, Viale dell’Università, 16, 35020 Legnaro, Italy; massimo.faccoli@unipd.it

**Keywords:** *Tomicus destruens*, Greece, mtDNA, super cooling point

## Abstract

*Tomicus destruens* (Wollaston), the Mediterranean pine shoot beetle, has long been confused with *Tomicus piniperda* (Linnaeus) in Greece, as well as in many other Mediterranean countries. To clarify its presence and population structure, we analyzed COI sequences of specimens from 21 Greek sites (including islands) and compared them with samples from central (Italy) and western (Spain) Mediterranean populations. Greek populations were dominated by a single haplotype (Td06), whereas Samothraki Island harbored several private haplotypes, indicating long localized lineage persistence. In contrast, Italian and Spanish populations showed high haplotype diversity with many region-specific lineages, consistent with long-term refugial stability in the central-western Mediterranean. Physiological measurements further differentiate the two species: the mean supercooling point (SCP) of Greek *T. destruens* (–12.3 °C) was substantially higher than reported for *T. piniperda* (≈–18 °C), demonstrating lower cold tolerance. This supports its restriction to mild Mediterranean climates. Overall, our results confirm the dominant occurrence of *T. destruens* with no recovery of *T. piniperda* individuals in Greece and highlight the combined value of genetic and physiological markers for resolving species boundaries and understanding Mediterranean bark beetle biogeography.

## Simple Summary

*Tomicus destruens* is a Mediterranean bark beetle species that attacks pine trees and can cause serious ecological and economic damage in forest ecosystems. In Greece, this species has long been confused with the closely related *Tomicus piniperda*, making its true distribution uncertain. The aim of this study was to confirm the presence of *T. destruens* in Greece and to investigate its genetic diversity and cold tolerance in comparison with populations from Italy and Spain. Adult beetles were collected from 18 locations across Greece, and mitochondrial COI gene sequences were analyzed to identify haplotypes and phylogeographic relationships. The results showed that most Greek populations shared one dominant haplotype, while several unique haplotypes were restricted to Samothraki Island, suggesting long-term isolation and local diversification. In contrast, Italian and Spanish populations exhibited much higher haplotype diversity. The study also examined the supercooling point (SCP) of *T. destruens*, a physiological indicator of cold tolerance. Greek populations showed higher SCP values than *T. piniperda*, indicating lower cold resistance and adaptation to milder Mediterranean climates. Together, the genetic and physiological findings confirm the presence of *T. destruens* in Greece and help explain its current geographic distribution and evolutionary history.

## 1. Introduction

*Tomicus destruens* (Wollaston, 1865) is a phloeophagous bark beetle with significant ecological and economic importance in Mediterranean pine ecosystems. It predominantly infests Mediterranean pine species such as *Pinus halepensis*, *P. brutia*, *P. pinaster*, *P. canariensis*, and *P. pinea*, although infestations have also been recorded on exotic pine species (*P. radiata*) and occasionally on *P. nigra* in Turkey [1], Spain [2], and Italy [3]. Infestations of *T. destruens* often follow periods of climatic stress, particularly at low-elevation, drought-prone forests across southern Europe and northern Africa [3,4]. Furthermore, outbreaks may contribute significantly to tree decline and mortality, especially in combination with other biotic and abiotic stressors [5].

Due to their morphological similarity and the partial overlap of their host and distribution ranges, *T. destruens* has historically been misidentified as *Tomicus piniperda* (L.) or considered a reddish Mediterranean form of it (*Blastophagus piniperda* var. *rubripennis* Reitter or *Blastophagus piniperda* var. *rubescens* Krausse). However, accurate morphological comparison [6] and molecular analyses [7,8,9] have clarified their taxonomic status as distinct species. Moreover, the use of mitochondrial (COI, COII) and nuclear (ITS1, ITS2) markers has enabled accurate species delimitation and revealed significant intraspecific genetic structure in *T. destruens* populations across its distribution range with distinct geographically associated haplotypes that suggested limited gene flow and possible local adaptations [7,10,11]. Nuclear ITS markers have also proven to be useful for diagnostic purposes, further supporting the species’ validity [8]. Even though *T. destruens* has been extensively reported in several Mediterranean countries [12], its occurrence and distribution in Greece remains doubtful and is scarcely and rather insufficiently documented. Historical records are often ambiguous due to the lack of molecular verification and strong morphological similarity with other *Tomicus* species, such as *T. piniperda*. To date, no published studies have examined the genetic structure of potential *T. destruens* populations in Greece, leaving a gap in the species’ phylogeographic framework.

In the context of physiological adaptation, cold tolerance plays a crucial role in the overwintering survival and geographic distribution of insect species, bark beetles included [13,14]. The supercooling point (SCP), defined as the temperature at which body fluids spontaneously freeze [15], serves as a key physiological trait in understanding the thermal tolerance of insect species and can thus be used as an additional species-specific diagnostic trait [16]. Studies on *T. piniperda* have shown that it possesses a relatively low SCP (i.e., high resistance to low temperatures) assessed around −18 °C for adults and −12.5 °C for larvae [17,18]. This is consistent with its natural distribution up to the higher European latitudes and with its overwintering in litter or insulated sites rather than exposed bark [13]. By contrast, *T. destruens*, confined to the Mediterranean basin, is likely to exhibit higher SCP, adapted to milder winters that facilitate its typical reproduction from November to March. Consequently, cold tolerance metrics like SCP can serve as an additional informative tool in delimiting species boundaries and understanding their environmental adaptability, particularly in regions where two species may co-occur, like *T. destruens* and *T. piniperda* in Europe [2,19] or *T. minor* (Hartig) and *T. yunnanensis* (Kirkendall and Faccoli) in China [20].

Although *T. destruens* has been extensively studied across western Mediterranean regions, its distribution and physiological adaptation throughout the southeastern Mediterranean regions remain unresolved. This study aims to provide the first phylogeographic analysis of *T. destruens* in southeastern Europe, combining phylogenetic and demographic data with physiological (SCP) traits. Additionally, our work attempts to resolve the taxonomic uncertainty concerning the occurrence and distribution of *T. destruens* in Greece, understanding the species’ ecological adaptability, something that has direct implications for the improvement in forest pest monitoring and management in Mediterranean pine ecosystems.

## 2. Materials and Methods

### 2.1. Insect Sampling

**Figure 1:**
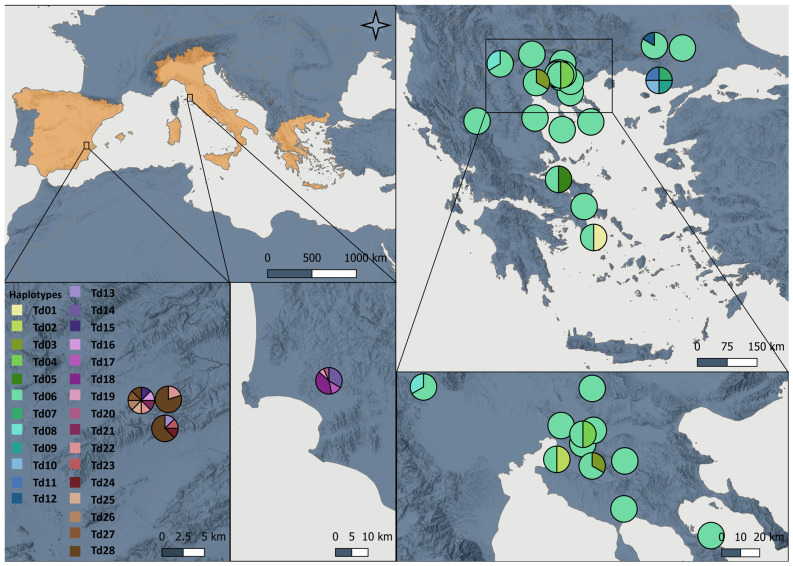
Maps showing the populations screened, and the haplotypes retrieved, identified by different colors (Td1–Td12: light green to dark blue for Greek haplotypes; Td13–Td28: purple to brown for Italian and Spanish haplotypes).

Between 2019 and 2020, adult *Tomicus* specimens were individually and manually collected from shoots of infested pine trees (*Pinus* spp.—Table 1) at 21 locations across Greece (Figure 1). A total of 83 alive adults were sampled and individually preserved in ethanol (≥70%) before being stored at −20 °C at the Forest Research Institute laboratory. Additionally, *T. destruens* specimens from Italy (Follonica, Tuscany) and Spain (Valencia) were included in the analysis to verify the taxonomic identity of Greek samples and examine their phylogenetic relationship.

### 2.2. Insect Molecular Analysis

Genomic DNA was extracted using the PureLink^®^ Genomic DNA Kit (Invitrogen, Waltham, MA, USA), following the manufacturer’s protocol, with the only modification being the use of stainless-steel grinding balls for tissue homogenization. PCR amplifications targeted a ~850 bp fragment of the mitochondrial COI gene that is broadly used in DNA barcoding, using primers 2183 (5′-caa cat tta ttt tga ttt ttt gg-3′) and 3041 (5′-tcc aat gca cta atc tgc cat att a-3′) [21], in 25 μL reaction volumes. Each reaction contained 8 μL of genomic DNA, 10.6 μL of double-distilled water, 5 μL of Red Bioline buffer (included with the Taq), 0.5 μL of each primer, and 0.4 μL of MyTaq (Red Bioline, Thane, India), resulting in a total reaction volume of 25 μL. PCR cycling conditions followed standard protocols used in previous studies [22] and were as follows: initial denaturation at 94 °C for 3 min, followed by 40 cycles of 94 °C for 30 s (denaturation), 45 °C for 30 s (annealing), and 72 °C for 1.5 min (extension), with a final extension at 72 °C for 7 min. PCR products were purified using the PureLink^®^ PCR Purification Kit (Invitrogen). Sequencing was performed by CEMIA SA (Larissa, Greece) using an ABI 3730XL automated sequencer (Thermo Fischer Scientific, Waltham, MA, USA).

### 2.3. Phylogenetic Analysis

Chromatograms were visualized using Chromas Lite^®^ (Technelysium Pty Ltd., South Brisbane, QLD, Australia) and aligned with Clustal X v.2.1 [23]. Haplotypes represented by single specimens were confirmed by re-sequencing an independent PCR amplicon to avoid base misincorporation due to polymerase errors [24]. Confirmed haplotypes were submitted to GenBank. Standard molecular diversity indices, including haplotype diversity (Hd), nucleotide diversity (π) [25], and average nucleotide differences (k) [26], were calculated using MEGA version 12 [27]. Phylogenetic relationships were inferred via Bayesian analysis using MrBayes v3.1.1 [28]. The optimal nucleotide substitution model (TPM2uf+I) was determined using jModeltest v0.1.1 [29], which is based on “Phyml” [30] under the Akaike information Criterion (AIC). Bayesian run consisted of 5 × 10^6^ generations with sampling every 100 generations. Convergence was determined by stabilization of the standard deviation of split frequencies, and the first 25% trees were discarded as burn-in. In addition, phylogenetic inference was assessed using the Maximum Likelihood approach as this is implemented in MEGA version 12 [27] (Tamura-3 parameters distances, NNI ML heuristic method and 1000 bootstrap replicates). In all these analyses, two sequences of *T. piniperda* (KX035226 and OQ934109) were used as outgroups.

Finally, demographic history was investigated using Tajima’s D [31], Fu and Li’s F [32], and mismatch distribution analyses, all conducted in MEGA version 12 [27]. A statistical parsimony network was generated using ANECA [33], which relies on TCS [34] and GeoDis [35] to infer the demographic processes that created the observed pattern of divergence.

### 2.4. Super Cooling Point Analysis

Supercooling points (SCPs) were assessed to evaluate the cold tolerance of *T. destruens* adults following protocols described by Andreadis et al. [36], adapted for bark beetles. Ten adults from pine shoots were sampled in early, mid-, and late September 2019 (Ntot = 30), placed individually in 2 mL Eppendorf tubes, and immobilized with cotton. A copper-constantan thermocouple (Digitron 2000T, Kalestead Ltd., Braintree, UK) was attached to the thorax of each beetle to record body temperature. Tubes were then placed inside a glass test tube (1.5 cm in diameter × 17 cm in height), which was submerged in a refrigerated bath (Model 9505, PolyScience, Warrington, PA, USA) containing 1:1 ethylene glycol and water. Cooling rate was set at 1 °C min^−1^ starting from 20 °C. For each specimen, SCP was recorded as the lowest temperature reached before the exothermic release of latent heat. To minimize the presence of ice-nucleating gut bacteria, specimens were kept in aerated plastic containers (5 cm in diameter × 8 cm in height) covered with nylon mesh and starved for 5–6 h before testing [37] Differences between means of SCPs observed at each of the three sampling periods were compared by one-way ANOVA using IBM SPSS Statistics (Version 29).

## 3. Results

### 3.1. Phylogenetic Analysis of Tomicus Haplotypes

**Figure 2:**
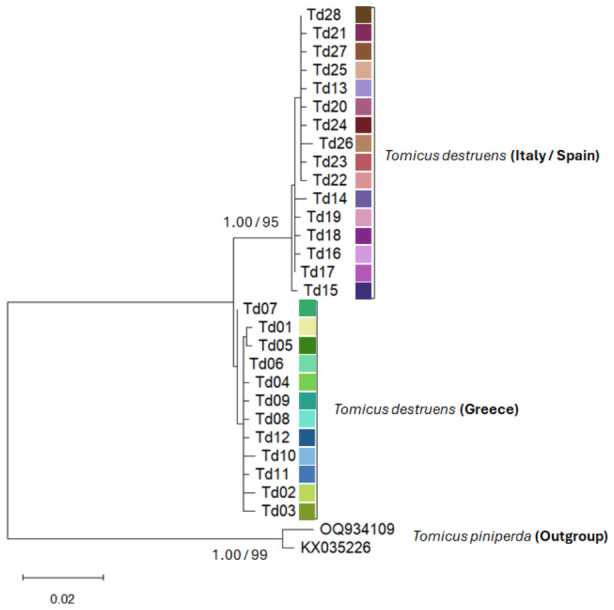
Rooted 50% majority rule consensus tree derived from the Bayesian analysis of *Tomicus destruens* haplotypes from Greece, Italy, and Spain. Values above branches indicate (a) Bayesian posterior probabilities and (b) Maximum Likelihood bootstrap support values (a/b). The scale bar shows the estimated number of substitutions per nucleotide. Haplotypes are assigned to colors as shown in Figure 1. *T. piniperda* sequences (KX035226 and OQ934109) are used as outgroup.

**Figure 3:**
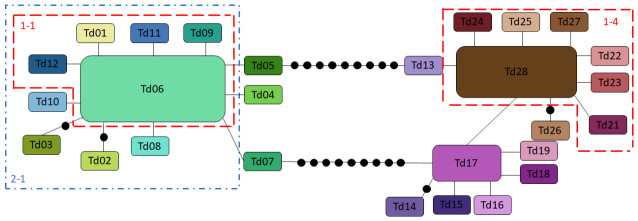
*Tomicus destruens* haplotype network. Haplotypes are assigned to colors as shown in Figure 1, with black circles indicating missing haplotypes. Dotted lines highlight those nested clades of the analysis for which a statistically supported demographic inference was concluded.

**Figure 4:**
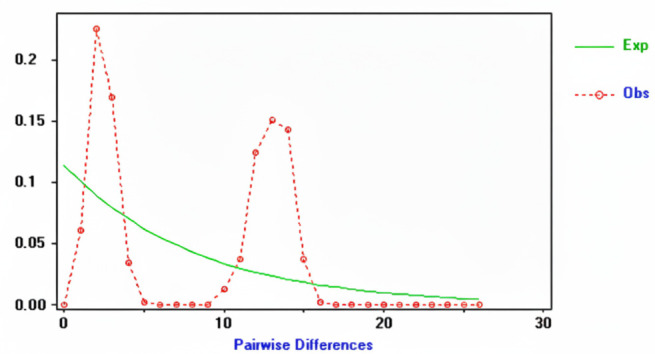
Mismatch distribution plot of the observed frequencies of pairwise differences among the retrieved haplotypes.

A total of 119 specimens of *Tomicus* sp. were sequenced from 21 populations in Greece (N = 83), one population from Italy (N = 15), and three populations from Spain (N = 21). Haplotype analysis revealed the presence of 28 distinct haplotypes (GenBank Accession numbers: PX854942–PX854969) across the sampled range (Table 2, Figure 1). Greek populations harbored 12 haplotypes (Td01–Td12), Italian samples contained five haplotypes (Td14, Td17–Td20), and Spanish samples included 11 haplotypes (Td13, Td15–Td16, Td21–Td28). The haplotype network was dominated by haplotype Td06, which occurred in most Greek localities, while several haplotypes were unique to single populations, particularly in insular or peripheral regions such as Samothraki (Td07–Td11). Greek haplotypes (Td01–Td12) exhibited low levels of divergence among them (1–4 substitutions), while larger distances were observed between Greek and western Mediterranean haplotypes (12–16 substitutions), evidence of a distinct geographic partitioning between haplotype groups. Both phylogenetic approaches (ML and Bayesian analysis) produced a well-resolved tree with *T. piniperda* as the outgroup species that clearly highlights the monophyly of *T. destruens* (Figure 2). All Greek haplotypes clustered together in a single clade (Td01–Td12), which was clearly separated from Italian and Spanish *T. destruens* haplotypes (Td13–Td28). The separation between Greek and Italian/Spanish *T. destruens* haplotypes was further depicted in the NCPA network, as these two clusters are separated by nine mutational steps (Figure 3). Phylogenetic inference identified Clade 1-1 and Clade 1-4 as cases of contiguous range expansion, while Clade 2-1 indicated restricted gene flow with isolation by distance. Mismatch distribution analysis produced a multimodal curve (Figure 4), consistent with population stability or structure. Neutrality tests yielded a negative but non-significant Tajima’s D value (D = –0.313, *p* > 0.01), suggesting no evidence of strong recent demographic expansion.

### 3.2. Super Cooling Point

**Figure 5:**
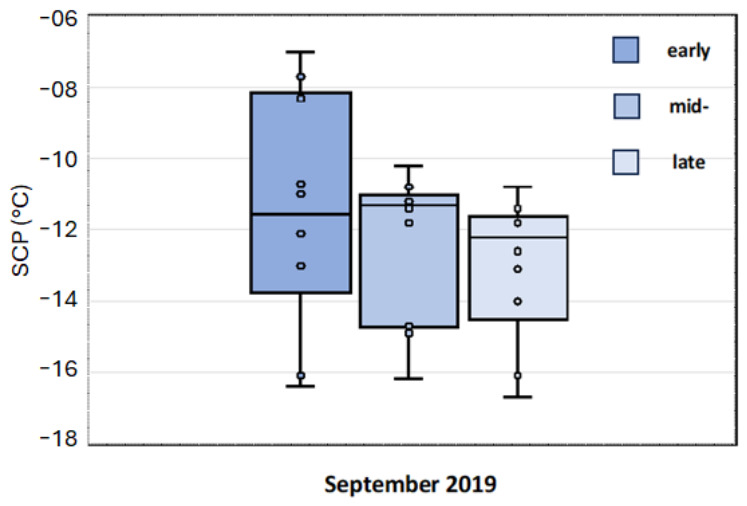
Supercooling points (SCP) of field-collected adults of *Tomicus destruens* (collected on 1, 15, and 30 September 2019). Each point represents the mean ± SE (*n* = 10).

Finally, cold tolerance assays performed on 30 Greek adults of *T. destruens* revealed an SCP (mean ± S.E.) of –12.3 ± 0.5 °C. When adults were tested across the three different sampling periods, the cold tolerance SCP values (mean ± S.E.) increased over time with –11.4 ± 1.0 °C in early September, –12.2 ± 0.7 °C in mid-September, and –13.0 ± 0.6 °C in late September (Figure 5). Although the SCP showed a gradual decrease over time, these differences were not statistically significant (*p* > 0.05), indicating a stable cold tolerance in *T. destruens* during the sampling period.

## 4. Discussion

### 4.1. Tomicus destruens Occurrence in Greece

Our study provides the first comprehensive phylogeographic analysis of *T. destruens* in southeastern Europe, addressing a relatively long-standing taxonomic ambiguity regarding its occurrence in Greece. Historically, populations from the Balkans have often been reported solely as *T. piniperda* [38], largely due to the high degree of morphological similarity between the two species, particularly in external adult characters [6,7,39]. For instance, ref. [40] erroneously reported only *T. piniperda* along the Ionian coast of Italy, although this species does not occur in these regions, as current studies have shown [41]. The overlap in hosts and habitats further contributed to this confusion, with many authors treating Greek and Turkish populations as *T. piniperda* [42,43]. Our results, integrating phylogenetic reconstruction, haplotype distribution, and cold-hardiness physiology, provide unequivocal evidence that all Greek specimens we examined within the sampled localities belong to *T. destruens*. This clarification is significant, as it robustly verifies the confirmed eastern range limit of *T. destruens* into the Balkans and indicates that the species’ distribution and populations may have been underestimated in earlier studies. The hypothesis that *Tomicus destruens* and *T. piniperda* merely represent two molecular or ecological forms of a single species is not supported by the available evidence. As summarized by Lieutier and colleagues [12], these two taxa differ consistently in stable diagnostic morphological traits (e.g., declivital interstriae, antennal coloration, body proportions, and protibial dentition [6]) that remain valid even in areas where species coexist. Additionally, persistence of distinct genetic lineages in sympatric areas strongly argues against conspecificity, since ongoing gene flow would homogenize populations. Instead, all available evidence supports reproductive isolation and validates *T. destruens* as a distinct Mediterranean species rather than just an ecological form of *T. piniperda*. Similar cases of cryptic and mistaken identity are widespread in Scolytinae [44], and molecular data have repeatedly proven indispensable for taxonomic resolution. Correct species identification is crucial, since *T. destruens* and *T. piniperda* differ not only in physiology but also in their phenology, outbreak dynamics, and responses to management strategies [45,46]. Misidentification can thus lead to ineffective control measures and erroneous ecological interpretations. By resolving this ambiguity, our study not only contributes to the biogeography and systematics of the genus *Tomicus* but also has direct applied significance for forest health monitoring and pest management in southeastern Europe.

### 4.2. Tomicus destruens Phylogeography

The Maximum Likelihood tree revealed strong geographic partitioning between *T. destruens* haplotypes from Greece (Td1–Td12) and those from Italy and Spain (Td13–Td28). This east–west separation is highly consistent with patterns observed in many other Mediterranean taxa, including both insects and forest trees, where glacial refugia preserved isolated lineages during Pleistocene climatic oscillations [47,48,49]. Our findings echo phylogeographic studies on *Ips typographus* (L.) [50] and *Thaumetopoea pityocampa* Denis & Schiffermüller [51], which also revealed east–west breaks corresponding to Balkan versus Iberian refugia. The strong bootstrap support of the clades reinforces the depth of these divergences. In *T. destruens*, this structuring indicates long-term isolation of Balkan populations, with limited or no recent gene flow to western Mediterranean lineages. The Mediterranean basin, with its complex topography and repeated glacial contractions, has long been recognized as a hotspot of phylogeographic structure, and our results confirm that *T. destruens* conforms to this general pattern [52,53].

The geographic distribution of haplotypes provides further evidence of phylogeographic structuring. In Greece, haplotype Td06 is dominant, occurring in nearly all sampled mainland populations, whereas private haplotypes (Td07–Td12) are solely restricted to Samothraki Island. Such patterns suggest that Td06 may represent a successful lineage that expanded from a Balkan refugium following glacial retreat, while the island haplotypes represent relictual diversity maintained and survived in local microrefugia [54,55]. This interpretation aligns with the “refugia-within-refugia” model [56] that emphasizes the role of insular and topographically complex sites in the preservation of unique haplotypes. In contrast, Italian and Spanish populations exhibited strikingly high haplotype diversity, with many haplotypes restricted to single populations (e.g., Td15–Td27). This pattern reflects the Iberian Peninsula’s well-documented role as a major glacial refugium, where multiple lineages coexisted and persisted through climatic oscillations [57,58]. On the other hand, Italian populations displayed an intermediate pattern, with multiple haplotypes shared among sites but also several locally restricted, suggesting a balance between historical gene flow and localized lineage persistence. Thus, the haplotype distribution data point to contrasting demographic histories: relative homogeneity and dominance of one lineage in Greece versus high lineage diversity in Spain and Italy.

### 4.3. Nested Clade Analysis

The Nested Clade Phylogeographic Analysis (NCPA) provided further resolution on the evolutionary processes shaping haplotype distributions. The nested cladogram separated Greek *T. destruens* haplotypes (Td01–Td12) from those in Spain and Italy (Td13–Td28) by nine mutational steps, confirming the deep divergence between eastern and western Mediterranean lineages. The inferences derived from the analysis highlight different processes acting at different hierarchical levels. For instance, clade 1–1 (containing Td01, Td06, Td09, Td11, Td12) and clade 1–4 (Td21–Td28) were both explained by contiguous range expansion, suggesting that once established, these lineages spread progressively into adjacent areas without major barriers to dispersal. In contrast, clade 2–1 (Td01–Td12) was interpreted as restricted gene flow with isolation by distance, indicating that even within Greece, dispersal is spatially limited, and genetic differentiation increases with geographic separation. These findings resonate with a broader phylogeographic theory: range expansion and isolation by distance often operate simultaneously in shaping genetic structure [59,60,61]. Similar patterns have been observed in bark beetles and other forest pests, where population structure reflects both expansion along host distributions and restricted dispersal due to landscape or ecological barriers [50,62,63]. While NCPA has sometimes been criticized for overinterpretation [64,65], in this case, its conclusions are strongly congruent with the phylogenetic tree and demographic results. By explicitly identifying contiguous expansion and isolation by distance, the analysis underscores the importance of both historical and contemporary processes in maintaining the east–west divergence of *T. destruens*.

Combining the multimodal mismatch diagram with the negative but not significant Tajima’s D neutrality test suggests neither strong recent expansion nor severe bottlenecking, but rather demographic stability or substructure. Such demographic patterns are common in populations that persisted within glacial refugia with limited subsequent expansion [66,67]. The dominance of Td06 across the Greek mainland, coupled with private haplotypes in Samothraki, is consistent with this model: a refugial core lineage expanded regionally, while isolated sites maintained unique haplotypes. Similar demographic stability has also been reported in other Mediterranean insects [51,62], supporting the view that southeastern Europe acted as a refugial zone with stable populations through Pleistocene climatic oscillations.

### 4.4. Super Cooling Point

Physiological traits provide an additional layer of interpretation. The mean SCP of Greek *T. destruens* was –12.3 °C, compared to approximately –18 °C reported for *T. piniperda* [68]. This difference is consistent with broader patterns observed in bark beetles: [13] demonstrated that species with lower SCPs generally exhibit greater cold tolerance and correspondingly broader or more northern geographic distributions. Recent work by [20] further confirmed that SCP, together with the accumulation of cryoprotective substances, is a reliable physiological indicator differentiating cold-tolerance strategies among *Tomicus* species. Thus, the higher SCP and reduced cold hardiness of *T. destruens* relative to *T. piniperda* fully align with its adaptation to milder Mediterranean winters and help explain the absence of this species from northern latitudes or higher elevations. Cold hardiness is widely recognized as a key determinant of insect distribution and persistence through climatic oscillations [45,69]. Therefore, the physiological limitation of *T. destruens* provides a mechanistic basis for its phylogeographic pattern—persisting and diversifying in southern refugia while failing to expand northward.

The broader phylogeographic scenario for *T. destruens* fits within the well-documented paradigm of multiple refugia in the Mediterranean basin. The Balkans, Iberia, and Italy have long been recognized as the three major European glacial refugia [47,48,49], and our data reinforce this model by showing distinct lineages in each region. However, beyond these large-scale refugia, our results also emphasize the role of microrefugia in shaping intraspecific diversity. The occurrence of unique haplotypes on Samothraki Island (Td07–Td12) suggests that small, geographically isolated habitats provided stable conditions for persistence even during adverse climatic periods. The concept of microrefugia—small, environmentally buffered sites maintaining populations outside the main refugial areas—is increasingly recognized as central to Mediterranean biogeography [52,54,70]. For insects with limited natural dispersal capacity, such as bark beetles, microrefugia may be particularly important, allowing lineages to survive at fine scales and later contribute to regional recolonization dynamics. Comparable evidence exists in other forest insects such as *T. pityocampa* [51] and *I. typographus* [63], where insular and mountainous refugia preserved distinct haplotypes. In the case of *T. destruens*, the coexistence of a dominant, widespread haplotype (Td06) with rare, localized haplotypes reflects a dual refugial dynamic: large-scale persistence in Balkan core refugia and fine-scale survival in microrefugia. This duality underscores the complexity of the Mediterranean landscape and phylogeography, where species’ histories are shaped not only by continental-scale glacial refugia but also by the fine-grained heterogeneity of the landscape that buffered populations through climatic oscillations and continues to maintain diversity under present-day climatic variability.

In conclusion, the integration of phylogenetic and demographic data with physiological traits provides a coherent phylogeographic narrative for *T. destruens*. Greek populations form a distinct, cohesive lineage, shaped by long-term persistence in a Balkan refugium, limited dispersal, and adaptation to milder climates. On the other hand, Spanish and Italian populations reflect a complex lineage diversity, as evinced in previous studies [10,11]. These results fit within the wider framework of Mediterranean phylogeography, where multiple refugia and microrefugia contributed to present-day diversity [53,58]. Beyond evolutionary insights, our findings can have applied significance, as distinct lineages might differ in host use and expanding potential with direct implications for forest pest management [46]. Additionally, accurate lineage identification may improve early-warning and monitoring programs by enabling the prediction of population responses to climatic warming, invasion risk, and host susceptibility across Mediterranean pine ecosystems. Future research should expand sampling into Anatolia and Northwestern Africa, apply genomic tools for higher resolution, and integrate ecological niche modeling to test hypotheses of climatic adaptation and range dynamics under future warming scenarios.

## Figures and Tables

**Table 1:** Population data (Location, Country, Coordinates, Host tree) and specimens screened per population.

Location	Country	X	Y	No. of Specimens	Host Tree
Rodopi	Greece	41°10′14.55″ N	25°28′50.83″ E	6	*P. brutia*
Moudania (Halkidiki)	Greece	40°14′42.70″ N	23°16′45.33″ E	2	*P. halepensis*
Lagkadas	Greece	40°49′18.76″ N	23°5′0.01″ E	3	*P. brutia*
Thermi (Thessaloniki)	Greece	40°32′57.36″ N	23°1′33.93″ E	2	*P. brutia*
Lagonisi	Greece	37°46′41.62″ N	23°53′25.78″ E	2	*P. halepensis*
Galatista (Halkidiki)	Greece	40°28′7.10″ N	23°17′3.91″ E	1	*P. brutia*
Hortiatis	Greece	40°36′39.60″ N	23°5′31.24″ E	1	*P. brutia*
Samothraki	Greece	40°29′8.36″ N	25°36′11.10″ E	4	*P. brutia*
Kilkis	Greece	41°0′3.56″ N	22°52′42.98″ E	8	*P. brutia*
Orhomenos	Greece	38°29′40.25″ N	22°58′16.35″ E	2	*P. brutia*
Thessaloniki	Greece	40°37′57.41″ N	22°58′33.41″ E	4	*P. brutia*
Kassandra (Halkidiki)	Greece	40°1′56.24″ N	23°24′41.51″ E	8	*P. halepensis*
Edessa	Greece	40°48′43.14″ N	22°2′51.46″ E	3	*P. brutia*
Parthenonas (Halkidiki)	Greece	40°7′12.75″ N	23°48′52.66″ E	3	*P. halepensis*
Paralia (Thessaloniki)	Greece	40°36′8.88″ N	22°56′58.55″ E	4	*P. pinea*
Panorama (Thessaloniki)	Greece	40°35′31.97″ N	23°1′37.64″ E	4	*P. brutia*
Vassilika	Greece	40°30′30.26″ N	23°4′59.22″ E	6	*P. brutia*
Dadia	Greece	41°7′23.24″ N	26°12′50.31″ E	5	*P. brutia*
Egaleo	Greece	38°0′20.79″ N	23°38′27.97″ E	6	*P. halepensis*
Ioannina	Greece	39°40′18.55″ N	20°50′13.62″ E	5	*P. brutia*
Agia	Greece	39°44′4.48″ N	22°42′4.79″ E	4	*P. brutia*
Follonica—Tuscany	Italy	42°57′25.79″ N	10°45′28.31″ E	15	*P. pinea*
Valencia T14	Spain	38°45′4.87″ N	0°45′49.12″ W	5	*P. halepensis*
Valencia T33	Spain	38°44′59.71″ N	0°46′6.07″ W	8	*P. halepensis*
Valencia Bloque 1	Spain	38°45′6.36″ N	0°45′57.08″ W	8	*P. halepensis*

**Table 2:** Number of specimens screened per population, and haplotypes retrieved. The number of individuals of each haplotype is given in parentheses (“Td” stands for *Tomicus destruens* haplotypes).

Location	No. of Specimens	No of Haplotypes	Haplotypes
Rodopi	6	2	Td12(1); Td06(5)
Moudania (Halkidiki)	2	1	Td06(2)
Lagkadas	3	1	Td06(3)
Thermi (Thessaloniki)	2	1	Td06(2)
Lagonisi	2	2	Td01(1); Td06(1)
Galatista (Halkidiki)	1	1	Td06(1)
Hortiatis	1	1	Td06(1)
Samothraki	4	4	Td07(1); Td09(1); Td10(1); Td11(1)
Kilkis	8	1	Td06(8)
Orhomenos	2	2	Td05(1); Td06(1)
Thessaloniki	4	1	Td06(4)
Kassandra (Halkidiki)	8	1	Td06(8)
Edessa	3	2	Td06(2); Td08(1)
Parthenonas (Halkidiki)	3	1	Td06(3)
Paralia (Thessaloniki)	4	2	Td02(2); Td06(2)
Panorama (Thessaloniki	4	2	Td04(2); Td06(2)
Vassilika	6	2	Td03(2); Td06(4)
Dadia	5	1	Td06(5)
Egaleo	6	1	Td06(6)
Ioannina	5	1	Td06(5)
Agia	4	1	Td06(4)
Follonica—Tuscany	15	5	Td14(5); Td17(2); Td18(6); Td19(1); Td20(1);
Valencia T14	5	2	Td22(1); Td28(4)
Valencia T33	8	8	Td15(1); Td16(1); Td21(1); Td22(1); Td25(1); Td26(1); Td27(1); Td28(1)
Valencia Bloque 1	8	4	Td13(1); Td23(1); Td24(1); Td28(5)

## Data Availability

Sequences obtained in the current study are given the following Accession Numbers: PX854942-PX854969, and will be made available in NCBI GenBank after the 1 January 2027.
